# Neurocognitive Function in HIV-Infected Patients: Comparison of Two Methods to Define Impairment

**DOI:** 10.1371/journal.pone.0103498

**Published:** 2014-07-31

**Authors:** Alejandro Arenas-Pinto, Alan Winston, Wolfgang Stöhr, John Day, Rebecca Wiggins, Say Pheng Quah, Jonathan Ainsworth, Sue Fleck, David Dunn, Alex Accoroni, Nicholas I. Paton, for the PIVOT Trial Team

**Affiliations:** 1 MRC Clinical Trials Unit at UCL, Institute of Clinical Trials and Methodology, London, United Kingdom; 2 Centre for Sexual Health and HIV Research, University College London, London, United Kingdom; 3 Section of Infectious Diseases, Division of Medicine, Imperial College, St Mary’s Hospital Campus, London, United Kingdom; 4 Department of HIV and GU Medicine, Imperial College Healthcare NHS Trust, London, United Kingdom; 5 Infectious Diseases, Southend University Hospital NHS Foundation Trust, Westcliff-on-Sea, United Kingdom; 6 Centre for Immunology and Infection, Department of Biology, University of York, York, United Kingdom; 7 Department of GU Medicine, Royal Victoria Hospital, Belfast, United Kingdom; 8 Infectious Diseases, North Middlesex University Hospital, London, United Kingdom; 9 Psychology and Psychotherapy Department, The Mortimer Market Centre, Central and North West London NHS Foundation Trust, London, United Kingdom; 10 Yong Loo Lin School of Medicine, National University of Singapore, Singapore; University of Glasgow, United Kingdom

## Abstract

**Objective:**

To compare two definitions of neurocognitive impairment (NCI) in a large clinical trial of effectively-treated HIV-infected adults at baseline.

**Methods:**

Hopkins Verbal Learning test-Revised (HVLT-R), Colour Trail (CTT) and Grooved Pegboard (GPT) tests were applied exploring five cognitive domains. Raw scores were transformed into Z-scores and NCI defined as summary NPZ-5 score one standard deviation below the mean of the normative dataset (i.e. <−1SD) or Z-scores <−1SD in at least two individual domains (categorical scale). Principal component analysis (PCA) was performed to explore the contribution of individual tests to the total variance.

**Results:**

Mean NPZ-5 score was −0.72 (SD 0.98) and 178/548 (32%) participants had NPZ-5 scores <−1SD. When impairment was defined as <−1SD in at least two individual tests, 283 (52%) patients were impaired. Strong correlations between the two components of the HVLT-R test (learning/recall) (r = 0.73), and the CTT and (attention/executive functioning) (r = 0.66) were observed. PCA showed a clustering with three components accounting for 88% of the total variance. When patients who scored <−1SD only in two correlated tests were considered as not impaired, prevalence of NCI was 43%. When correlated test scores were averaged, 36% of participants had NPZ-3 scores <−1SD and 32% underperformed in at least two individual tests.

**Conclusion:**

Controlling for differential contribution of individual test-scores on the overall performance and the level of correlation between components of the test battery used appear to be important when testing cognitive function. These two factors are likely to affect both summary scores and categorical scales in defining cognitive impairment.

**Trial registration:**

EUDRACT: 2007-006448-23 and ISRCTN04857074.

## Introduction

HIV-associated neurocognitive impairment (NCI) remains a major issue in the care of chronic HIV management. Cognitive impairment is still common, even among patients successfully treated with anti-retroviral therapy (ART) [1], [2]. However controversy remains regarding the best way to, firstly, assess cognitive function and secondly, interpret cognitive function results in HIV-infected patients [3]–[5]. In research settings, a typical approach is to take the average of demographically adjusted scores on individual test domains to create a summary score as a marker of cognitive function and in some cases, to use this as markers to quantify cognitive impairment [2], [6], [7] but summary scores assume equivalent contribution of each test score on the overall summary score and do not offer differential information on the cognitive domain which may be affected. Other approaches such as the global deficit score have also been used to generate overall composite scores but in general, these do not offer differential information on the cognitive domain which may be affected [8], [9].

According to the 2007 revised research criteria for classifying HIV-associated NCI (often known as the Frascati criteria or the HIV-associated neurocognitive disorders (HAND) classification) cognitive impairment is defined as performance of at least 1 standard deviation (SD) below the mean of demographically adjusted normative datasets in at least two different cognitive domains [10].

High level of agreement between summary deficit scores and HAND classification results has been reported but the latter often identified a larger number of impaired patients as compared to summary score deficits [10], [11]. This excess in diagnosis of impairment usually correspond with mild forms of the condition which may correspond to false positive results [3], [5]. The aim of this study was to compare these two methods to assess cognitive function in a large population of effectively treated HIV-infected adult individuals. In addition, we aimed to assess acceptability and suitability of a screening test battery exploring five cognitive domains in the context of a large randomised clinical trial (RCT).

## Methods

### Participant selection

This analysis used baseline data from individuals recruited into a large treatment strategy trial for long-term management of chronic HIV infection – the Protease Inhibitor monotherapy Versus On-going Triple-therapy (PIVOT) trial. The trial enrolled participants receiving a stable combination anti-retroviral therapy (cART) regimen and a plasma HIV RNA <50 copies/ml at screening and for at least 24 weeks prior to study entry. 20% of the study population had a history of an AIDS-defining condition. Participants were recruited between November 2008 and July 2010 and followed-up until November 2013.

The PIVOT study is registered as EUDRACT: 2007-006448-23 and ISRCTN04857074. National Research Ethics Service (NRES) approval for the trial, including the NC assessments, was obtained from the East of England Cambridge South Ethics Committee. Written informed consent was obtained from all study participants.

### Neurocognitive testing

Neurocognitive testing was performed by designated research staff at each study site after receiving appropriate training by the coordinating centre. The training procedures included a face-to-face session, a training video and practice of the tests with at least five work colleagues before being allowed to assess study participants, followed by yearly revision. Five cognitive domains were explored with three different tests: Verbal learning and memory were assessed using *Hopkins Verbal Learning Test-Revised* (HVLT-R) [12], fine motor skills assessed using *Grooved Pegboard* [13], and attention and executive function assessed using *Color Trails Tests* (CTT) 1 and 2 respectively [14]. Neurocognitive function was tested on all study participants at baseline and these results have been reported elsewhere, but briefly 32% and 52% of subjects showed summary deficit scores and HAND-like classification results compatible with cognitive impairment when standard normative data provided by the test manufacturers were used [11].

Test performance was considered not valid if participants decided to abandon the test before completion, in the case of investigators failing to comply with standard procedures according to the instructions or if the test was interrupted because of external factors. In addition, all scores were centrally monitored and extreme results were investigated and excluded if considered to be related to any of the situations listed above.

### Statistical analysis

Only participants with complete cognitive testing results available were included in the analyses. Raw scores for each cognitive test were transformed to *z*-scores using the manufacturers’ normative data [12]–[14] adjusted for age (all tests) and years of education (CTT) by subtracting the mean and dividing by the standard deviation (SD) of test scores in reference populations. For the Grooved Pegboard test the z-score was obtained by taking the average of the z-scores for the dominant and non-dominant hands. Summary *z*-scores (NPZ-5) were then calculated by averaging z-scores of the 5 tests. For all individual test z scores and the NPZ-5, scores below zero denote below-average neurocognitive function compared to the reference population. Cognitive impairment was defined using two approaches: a) a summary NPZ-5 one SD below the mean of the normative dataset (i.e. < −1SD) (summary score) and b) test scores <−1SD in at least two individual tests (categorical scale).

Cochran’s Q test was used to ascertain heterogeneity of impairment across the individual tests. To investigate the relationship between all 5 tests, Pearson Correlation Coefficients were calculated. Principal component analyses (PCA), using correlation matrices, were performed to analyse the contribution of individual test scores, or tests scores clustering together, on the total variance of the 5 tests. First, a PCA without rotation and keeping all components was applied to get an overall picture. Principal components were orthogonal and uncorrelated. Second, rotated PCA using the varimax method was performed to reduce inter-component variance. Only those factors required to explain at least 80% of the total variance were retained in the rotated model which corresponded to 3 components based on the previous correlation analyses. Here, factors have only low or high values to facilitate interpretability of the components. Other rotation methods were also applied as was PCA using raw test scores instead of z-scores but results were very similar and therefore are not shown.

## Results

### Patient characteristics and acceptability of cognitive testing

Neurocognitive testing at baseline appeared to be highly acceptable. Of the 587 participants recruited into PIVOT, only one declined all tests. Overall, 548 (93%) participants produced valid results on all five tests and the proportion of missing, invalid or discontinued tests was not exceeding 2% in any of the tests except for the CTT-2 (4%). The demographic and clinical features of the 39 patients who were unable to produce complete tests did not differ from the general PIVOT population (Table 1).

**Table 1:** Baseline characteristics.

Gender, male	419 (76)
Age, years	44 (9)
Ethnicity	
Caucasian	375 (68)
Black	153 (28)
Other	20 (4)
Nadir CD4+, cells/µL	177 (118)
Baseline CD4+, cells/µL	554 (217)
Years undetectable HIV RNA	4 (3)
Years education	15 (4)
Years on cART	5 (3)
Hepatitis C antibody positive	20 (4)
Baseline haemoglobin, g/dL	14 (1)

Data are number (%) or mean (standard deviation).

### Measurement of neurocognitive function

**Figure 1:**
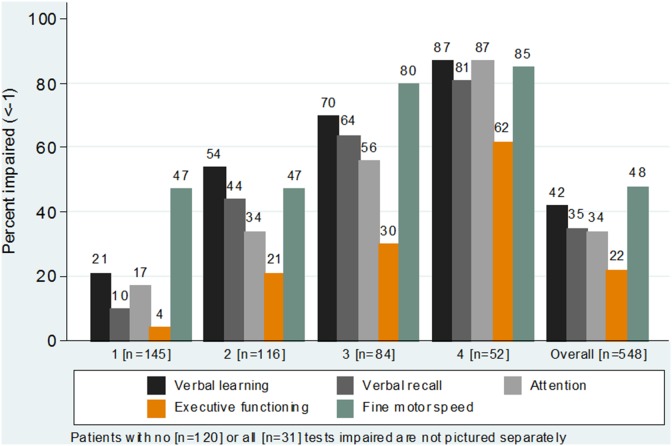
Proportion of patients with functional domains impaired (<−1SD), overall and by number of tests impaired.

Mean NPZ-5 score was −0.72 (SD 0.98) and 178/548 (32%) of the study participants had a NPZ-5 score <−1, compatible with cognitive impairment. Mean Z-score for all tests were below the normative average and ranged from −0.20 (SD 1.46) for the CTT-2 to −1.36 (SD 1.96) for the GPT scores (Table 2); similarly the proportion of participants having Z-scores <−1SD was lowest for the CTT-2 test (22%) and highest (48%) for the GPT test (p<0.001). When cognitive impairment was defined as <−1 in at least two individual tests (categorical scale) 283 (52%) patients were impaired and of those patients 116 (21%), 84 (15%), 52 (9%), and 31 (6%) were <−1SD on 2, 3, 4, and all 5 tests, respectively (Figure 1).

**Table 2:** Description of neurocognitive tests.

Test	Cognitive domain	Raw test value^¥^	z-score		impaired
		(median, IQR)	(mean, sd)	(median, IQR)	(z-score <−1)
HVLT-R learning	Verbal learning	25 (22, 29)	−0.70 (1.16)	−0.69 (−1.48, 0.12)	229 (42%)
HVLT-R recall	Verbal memory	9 (7, 11)	−0.58 (1.17)	−0.45 (−1.49, 0.38)	193 (35%)
Color Trail Test 1	Attention/speed of information processing	0∶43 (0∶34, 0∶56)	−0.74 (1.38)	−0.46 (−1.43, 0.27)	188 (34%)
Color Trail Test 2	Executive functioning	1∶21 (1∶05, 1∶42)	−0.20 (1.46)	0.11 (−0.87, 0.80)	118 (22%)
Grooved Pegboard Test^π^	Fine motor skills/complex perceptual	1∶17 (1∶09, 1∶27)	−1.36 (1.96)	−0.96 (−2.07, −0.17)	264 (48%)
NPZ-5^$^	Global z-score	-	−0.72 (0.98)	−0.52 (−1.23, −0.05)	178 (32%)

Notes: HVLT-R: Hopkins Verbal Learning Test–Revised; ^π^average of left and right hand; ^$^Average of the 5 tests; **^¥^**HVLT: words remembered correctly, other tests: min∶s. N = 548.

Overall agreement between the two definitions of impairment was 79%, with 261 (48%) and 174 (32%) being classified as both normal and both impaired, respectively. Of the remainder, 4 patients (0.7%) with NPZ-5 score <−1SD underperformed in only one test/domain (the GPT test in all cases). The other 109 (20%) patients had Z-scores <−1SD on at least two cognitive domains but had NPZ-5 scores within the normal range.

### Correlation between individual test Z-scores

27% of patients had z-scores <−1 on both HVLT-R learning and recall tests, 15% on HVLT-R learning but not recall test, and 8% on HVLT-R recall but not on learning test. 16% of patients had z-scores <−1 on both CTT-1 and CTT-2, 18% on CTT-1 but not CTT-2, and 5% in CTT-2 but not on CTT-1. Table 3 shows the correlation matrix of the 5 individual neurocognitive tests. There were strong correlations between a) HVLT-R learning and HVLT-R recall tests (r = 0.73), and b) CTT-1 and CTT-2 (r = 0.66). GPT was more correlated with the two CTTs (r = 0.33 with each) than with the two HVLT-Rs (r = 0.17 and 0.15) (Table 3).

**Table 3:** Correlation matrix of neurocognitive tests.

	HVLT-R learning	HVLT-R recall	CTT 1	CTT 2	GPT
HVLT-R learning	1				
HVLT-R recall	0.73	1			
CTT 1	0.23	0.19	1		
CTT 2	0.26	0.24	0.66	1	
GPT	0.17	0.15	0.33	0.33	1

Notes: correlation coefficients using test z-scores; HVLT-R: Hopkins Verbal Learning Test–Revised; CTT: Color Trail Test; GPT: Grooved Pegboard Test (average of left and right hand). Pearson Correlation Coefficient; p-value <0.001 for all correlations.

Inter-test correlations were further analysed using PCA. The first component explained 47% of the total variance and has positive loadings of similar size on all variables, so could be interpreted as overall neurocognitive functioning. The second principal component explains 26% of the total variance and has negative loadings of similar size on both parts of the HVLT-R and positive loadings for the other tests, therefore differentiating performance in HVLT-Rs versus CTT-1, CTT-2 and GPT. The third principal component similarly differentiates performance in both CTTs versus GPT and explains 15% of the total variance. The fourth and fifth principal component only explain about 7% and 5%, and differentiate performances in CTT-1 versus CTT-2, and between the 2 parts of the HVLT-R, respectively. PCA with rotation and restricted to 3 components showed a clustering similar to the results of the correlation analyses: component 1 consisted of the two HVLT-R tests, component 2 of CTT-1 and 2, and component 3 of the GPT (Table 4).

**Table 4:** Principal component analysis.

	Unrotated						Rotated			
	Component 1	Component 2	Component 3	Component 4	Component 5	unexplained variance	Component 1	Component 2	Component 3	unexplained variance
**Component** **loadings**										
HVLT-R learning	0.47	−0.52	0.02	0.08	−0.71	0	0.70	0.01	0.01	0.14
HVLT-R recall	0.45	−0.55	0.01	−0.03	0.70	0	0.71	−0.01	−0.01	0.13
CTT 1	0.47	0.42	−0.33	0.70	0.07	0	−0.02	0.71	0.01	0.17
CTT 2	0.49	0.38	−0.33	−0.71	−0.05	0	0.02	0.70	−0.01	0.17
GPT	0.35	0.31	0.88	−0.01	0.01	0	0.00	−0.00	1.00	<0.01
Eigenvalue	2.34	1.29	0.76	0.34	0.27		1.73	1.66	1.00	
% variance explained	0.47	0.26	0.15	0.07	0.05		0.35	0.33	0.20	
%, cumulative	0.47	0.73	0.88	0.95	1.00		0.35	0.68	0.88	

Notes: using test z-scores; HVLT-R: Hopkins Verbal Learning Test–Revised; CTT: Color Trail Test; GPT: Grooved Pegboard Test (average of left and right hand); Because a correlation matrix was analysed, the variables are standardized to have unit variance, so the total variance is 5.

Based on this finding, we recalculated cognitive impairment according to our categorical scale by reclassifying patients who underperformed only on two strongly correlated tests (HVLT-R both parts only (n = 36) or CTT-1 and 2 only (n = 14)) as ‘not impaired’. Prevalence of cognitive impairment would then be 43% rather than 52%. Similarly, when the two parts of the HVLT-R and CTT are averaged first and then used to calculate a NPZ-3 score with the GPT 36% of study participants showed NPZ-3<−1 whereas the proportion of participants with scores <−1 in two or three tests was 32%.

## Discussion

NCI is an important morbidity in HIV-infected populations and the Frascati criteria is the most widely used and accepted method of diagnosis and classification. However there are concerns about some possible limitations of this approach, particularly in the case of asymptomatic or milder forms of the condition [3]–[5]. In this group of virologically suppressed patients on cART we performed standard neurocognitive tests and explored the effect of using different approaches to analysis and classification of impairment. These approaches yielded differing results. When using summary NPZ-5 scores, calculated by averaging Z-scores for all explored cognitive domains, the proportion of cognitively impaired patients was 32%, whereas using a categorical scale (defined by poor performance on at least two domains) it was 52%.

Summary scores have frequently been used to assess cognitive function in HIV-infected patients [15]–[17] and other medical conditions [18]. They provide single numerical results, particularly useful for monitoring change in cognition over time and have been utilised in the context of an RCT exploring interventions to treat HIV-associated NCI [19]. However, with a summary score such as NPZ-5, all individual test scores contribute equally, which may not reflect their individual relationship to overall neurocognitive dysfunction. In our study, neurocognitive testing generated data where some variables were highly correlated which may suggest that a simple average of individual tests Z-scores may not be an optimal method to identify cognitive impairment. Normalising scores by transforming them into Z-scores adds another limitation, as available manufacturers’ normative data (obtained with populations of unknown HIV status) may not be entirely appropriate for comparison with HIV-infected populations [11], [20]. Other methods also based on average scores that are used to measure cognitive function by normalising raw-scores, such as the global deficit score (GDS) and the clinical rating system (CR), may not be affected by the limitations generated by the lack of suitable normative population datasets, but these do not take into consideration the level of correlation between functional domain scores. When compared head to head, GDS and CR also generated differing results with the CR system classifying as impaired a larger proportion of patients than GDS [8].

By using 1 SD below the normative mean to define impairment, and assuming normal distribution of the data, 16% of individuals with normal cognitive function are expected to be classified as impaired for a single test. In categorical evaluation systems such as the Frascati criteria the probability of underperforming in two or more cognitive domains will depend on the number of tests performed (the higher the number of tests, the higher the probability of obtaining two or more abnormal results) and the level of correlation between them (there would be a high probability of failing two or more highly correlated tests or domains) and therefore, the false positive rate increases with multiple testing and multiple measures in a complex fashion [3], [21]. Using a more strict cut-off (e.g. <−1.5 or 2 SD) to define impairment (and perhaps limiting the number of measures included on the testing battery to explore performance on key cognitive domains, to those with low inter-correlation) may provide a more accurate approach to identify cognitive impairment. However, using a stricter cut-off may decrease the sensitivity of the testing battery, thus increasing the rate of false negatives. We chose a cut-off of −1SD for the summary score to define impairment, however, it should be noted that the standard deviation of an average is smaller than single test scores (again depending on the correlation between individual tests) and, therefore, also the proportion below −1SD in a summary score compared with a single test.

PCA is a useful tool to reduce a large set of correlated scores into clusters of independent factors which could help in better understanding their contribution in the overall variance of the different scores. PCA generates weighted average scores which could be used summarise performance on cognitive testing batteries. Although the PIVOT battery was brief and involved only one test for each of the five cognitive domains explored, PCA showed scores clustering in an expected fashion in three independent factors which explained 88% of the overall variance. If patients in whom test scores were <−1 only in two domains clustering together in a single PCA factor were reclassified as ‘not impaired’, 43% of our study population could be considered as cognitively impaired, a prevalence lower than previously reported [22], [23]. The first component from our PCA un-rotated model showed similar loadings on all tests suggesting similar contribution on the overall variance (Table 4) and therefore, may support the appropriateness of using a simple average of test scores to summarise cognitive function. However, the GPT showed a somewhat smaller contribution reflecting its lower correlation with the other tests, and it could be suspected that the cognitive domain it measures has less weight in a simple average. As an alternative approach to measure cognitive function we averaged the two parts of the HVLT-R and CTT to calculate an NPZ-3 score. Here, we observed a greater level of agreement between the two definition of NCI as 36% and 32% of study participants showed NPZ-3 <−1 and scores <−1 in two or three tests, respectively.

Our study population was large, homogenous, effectively treated and derived from a multi-centre study which confers some strength to our findings. However, our testing battery was brief and by no means comprehensive. Using a larger battery may generate different prevalence of NCI even in the same population, perhaps increasing sensitivity. However, depending of the level of correlation between tests, including a larger number of observations may increase the probability of ≥2 abnormal results [3]. Simioni et al reported a very high prevalence (84%) of NCI in effectively suppressed patients using a very large battery and adjusting their analysis for multiple comparisons but not for correlation between the 39 scores used [22]. On the other hand, a number of short (2–4 tests) batteries exploring each cognitive domain with a single test have demonstrated good sensitivity (60–87%) and specificity (83–91%) compared to a comprehensive test battery [24].

Prevalence of NCI is highly dependent not only on the definition used, but also on normative datasets utilised for analysis since a number of socio-demographic and cultural factors might impact performance on neuropsychological tests. In our original analysis we used normative data provided by the manufacturers of each test as well as ethnicity-adjusted sets for the CTT and the HVLT-R to calculate prevalence of NCI [11], [25]. Large cross-sectional studies have used additional normative datasets corrected by factors such as education, gender and age to calculate NCI have reported prevalences ranging between 52–58%, similar to the prevalence reported by us when the manufactures’ dataset was used (52%) [26], [27]. Because the main aim of this analysis was explore the discordance between the calculated prevalence of NCI with two different definitions of impairment, we decided to use the same normative datasets used for our initial analysis [11]. Exploring the PIVOT battery and clustering of test scores using PCA in a cohort of HIV-negative individuals would generate some valuable data.

In summary, our analysis showed discrepancies between the two definitions of NCI with the categorical scale being more likely to identify a larger proportion of study participants as impaired compared to the summary score. Taking into consideration correlations between individual test scores and adjusting test scores with methods such as PCA may have greater overall neuropsychological validity.
